# Paramyxo- and Coronaviruses in Rwandan Bats

**DOI:** 10.3390/tropicalmed4030099

**Published:** 2019-07-02

**Authors:** Wanda Markotter, Marike Geldenhuys, Petrus Jansen van Vuren, Alan Kemp, Marinda Mortlock, Antoine Mudakikwa, Louis Nel, Julius Nziza, Janusz Paweska, Jacqueline Weyer

**Affiliations:** 1 Centre for Viral Zoonoses, Department of Medical Virology, Faculty of Health Sciences, University of Pretoria, Pretoria, Gauteng 0001, South Africa; 2 Centre for Emerging Zoonotic and Parasitic diseases, National Institute for Communicable Diseases, National Health laboratory Services, Sandringham, Johannesburg 2131, South Africa; 3 Rwanda Development Board, Department of tourism and Conservation, P.O Box 6239, Kigali, Rwanda; 4 Centre for Viral Zoonoses, Department of Biochemistry, Genetics and Microbiology, Faculty of Natural and Agricultural Sciences, University of Pretoria, Pretoria, Gauteng 0001, South Africa; 5 Mountain Gorilla Veterinary Project, P.O Box 115, Musanze, Rwanda

**Keywords:** paramyxovirus, coronavirus, Rwanda, bat, surveillance, caves, barcoding, henipavirus, jeilongvirus

## Abstract

A high diversity of corona- and paramyxoviruses have been detected in different bat species at study sites worldwide, including Africa, however no biosurveillance studies from Rwanda have been reported. In this study, samples from bats collected from caves in Ruhengeri, Rwanda, were tested for the presence of corona- and paramyxoviral RNA using reverse transcription PCR assays. Positive results were further characterized by DNA sequencing and phylogenetic analysis. In addition to morphological identification of bat species, we also did molecular confirmation of species identities, contributing to the known genetic database available for African bat species. We detected a novel *Betacoronavirus* in two Geoffroy’s horseshoe bats (*Rhinolophus clivosus*) bats. We also detected several different paramyxoviral species from various insectivorous bats. One of these viral species was found to be homologous to the genomes of viruses belonging to the *Jeilongvirus* genus. Additionally, a *Henipavirus*-related sequence was detected in an Egyptian rousette fruit bat (*Rousettus aegyptiacus*). These results expand on the known diversity of corona- and paramyxoviruses and their geographical distribution in Africa.

## 1. Introduction

Bats (Order Chiroptera) account for 20% of all mammalian species and are distributed worldwide. With the advancement in detection techniques and increased surveillance, bats are being increasingly recognized as hosts for many zoonotic viruses [1], including filo-, paramyxo-, corona- and lyssaviruses [2,3,4,5]. Regions in Africa are considered a hotspot for emerging infectious diseases with more than 50% of recently emerging diseases originating from wildlife species on this continent [6,7]. Although several surveillance studies have been implemented to detect potential zoonotic viruses in bats, including from countries in the Congo basin and East Africa, limited information is available for Rwanda. Importantly, in the bordering Democratic Republic of Congo and Uganda, Marburg and Ebola disease outbreaks in humans have occurred [8], and corona- and paramyxoviruses have been reported to circulate in bats [3,9,10,11]. 

Coronaviruses are positive-sense RNA viruses with the potential to cause respiratory, gastrointestinal, hepatic, and neurological diseases in their hosts [12] and are divided into four genera namely *Alphacoronavirus*, *Betacoronavirus*, *Gammacoronavirus*, and *Deltacoronavirus* [5]. Bats host a large diversity of coronaviruses and the expanding research can be largely attributed to the emergence of novel coronaviruses of public health and veterinary importance. Three such viruses emerged in the last 17 years, including the severe acute respiratory syndrome (SARS) coronavirus in 2002, Middle East respiratory syndrome (MERS) coronavirus in 2012, and the swine acute diarrhoea syndrome (SADS) coronavirus in 2017 [13,14]. 

Bat coronaviruses have been shown to be associated with particular bat genera and similar viruses have been identified throughout the geographical distribution of their hosts [11,12,15]. Diverse bat coronaviruses related to SARS coronavirus (now termed the *Sarbecovirus* subgenus) have been identified from the *Rhinolophus* bat genus (Horseshoe bats) in Asia, Europe and Africa [11,16,17]. Continued surveillance within these bats species in China identified lineages of recombinant SARS-related coronaviruses nearly identical to human SARS coronaviruses, capable of using the same receptor molecules [17,18,19]. As a result, these viruses have therefore been postulated to be capable of direct human infection [18,19]. Bats from various African countries, including Kenya, Ghana, Nigeria, Tanzania, Uganda and South Africa, have yielded a large diversity of novel coronaviruses [11,20,21,22,23,24,25,26,27]. Some of the bat coronaviruses identified have been shown to be genetically related to known human coronaviruses such as HCoV-229E, HCoV-NL63, and MERS coronavirus [11,21,24,25,26]. 

Paramyxoviruses are negative-sense single-stranded RNA viruses capable of infecting a diverse host range including mammals, birds, reptiles and fishes [28]. The taxonomic classification of viruses in the *Paramyxoviridae* family has recently undergone several changes [29]. In an attempt to accommodate the rapidly growing number of paramyxoviruses described, the previously known *Avulavirus* and *Rubulavirus* genera were elevated to the sub-family level (*Avula*- and *Rubulavirinae*) each with two new genera. In addition, several unclassified rodent-borne viruses were classified to newly established genera (*Narmovirus* and *Jeilongvirus*) in the sub-family *Orthoparamyxovirinae* to which the *Henipa-, Morbilli*- and *Respirovirus* genera belong. 

Several zoonotic paramyxoviruses have emerged as important public health threats in the past three decades. The emergence of Hendra and Nipah viruses (*Henipavirus* genus) during the 1990s in Australia and Southeast Asia respectively, marked the first report of zoonotic paramyxoviruses of considerable public health importance [30,31]. These viruses are characterized by high morbidity and mortality rates and outbreaks have been reported on a near annual basis. In addition, another paramyxovirus, Sosuga virus (*Pararubulavirus* genus), emerged as the etiological agent of a single non-fatal human infection contracted in Uganda, Africa [32]. The natural wildlife reservoir for these zoonotic viruses was determined to be the fruit bat species occurring in these areas, i.e., flying foxes from the *Pteropus* genus for the henipaviruses [33,34], and the Egyptian rousette bat (*Rousettus aegyptiacus*) for the rubulavirus [9]. Viruses related to the *Henipa*- and *Orthorubula*- and *Pararubulavirus* genera as well as a number of unclassified viruses have been described from countries bordering Rwanda as well as other African countries [3,9,10,35,36,37]. *R. aegyptiacus, Hipposideros* spp. and *Miniopterus inflatus* have tested positive for paramyxoviral RNA that is closely related to known human pathogens including the henipaviruses, human mumps virus, human parainfluenza virus 2 and human parainfluenza virus 4 [3,37].

In this study, we report the detection of a novel *Betacoronavirus* in *Rhinolophus clivosus* sampled in the Ruhengeri cave system in Rwanda. In addition, we report on the detection of *Jeilongvirus* and related sequences in *Hipposideros* spp. as well as a *Henipavirus*-related sequence in the fruit bat species *R. aegyptiacus*. These results expand on the known diversity of these virus groups and their geographical distribution in Africa.

## 2. Materials and Methods:

### 2.1. Study Area and Sample Collection

**Figure 1:**
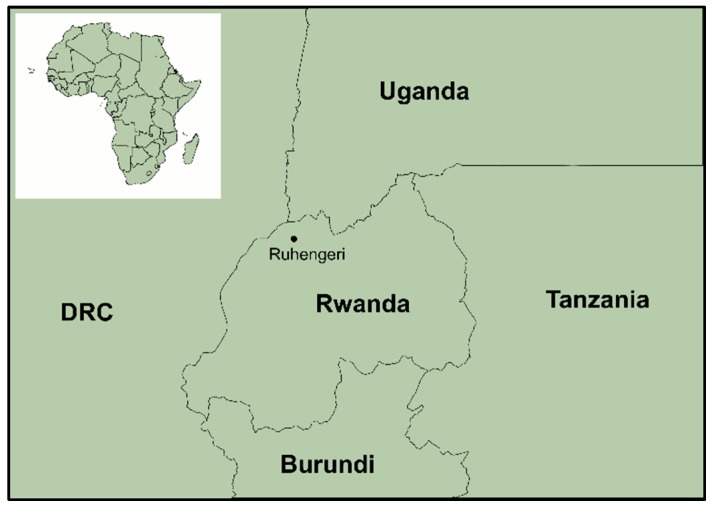
A map of Rwanda and bordering countries. The black dot indicates the site of the two caves in Ruhengeri where sampling was conducted. Map generated using the QGis software.

In December 2008, a team from the University of Pretoria, National Institute for Communicable Diseases, and Rwanda Tourism Board and National Park Authority visited two cave sites in Ruhengeri, Rwanda (GPS coordinates: 1°30′14.2″ S 29°37′59.9″ E; Figure 1) where bats were caught at night using mist nets in the surrounding areas, and two bank G4 Forest Strainer harp traps (Bat Conservation and Management, Inc., USA, Australia) at the cave entrances. When collecting samples in the field, personal protective equipment used included Tyvek suits (DuPont^TM^, Wilmington, DE, USA), disposable over gowns (Stylianou MediSupplies ltd, Middle East), 3M full powered air purifying respirators (3M, Maplewood, MN, USA), gumboots (Bata Industries^®^, Pinetown, South Africa), double layer nitrile gloves (Lasec, Cape Town, South Africa) and leather gloves (Evrigard, Johannesburg, South Africa). 

Bats were placed in individual cotton bags before processing. Bats were morphologically identified [38] and data including sex, reproductive status, forearm length and weight were also recorded. Samples collected from bats included fecal and oral swabs, wing biopsies in 70% ethanol and blood (serum) for use in viral surveillance studies. Oral swabs were collected by gently swabbing the inside of the mouth (cheeks and tongue) with a sterile swab (VWR Critical Swab, Atlanta, GA, USA). Fecal material or swabs (VWR Critical Swab, Atlanta, GA, USA) were collected from the bat or the cotton bag, when it was available. Sterile foreceps were used to collect fecal pellet(s) and place them in 2 mL microcentrifuge tubes (Sarstedt, Nümbrecht, Germany). Urine was collected with a sterile swab (VWR Critical Swab, Atlanta, GA, USA) from individual bats as was available. In instances where bats died during processing, necropsies were performed and various organs and tissues, including kidney, spleen, heart, pectoral muscles, liver, lung, stomach, bladder, tongue, brain and lymph nodes, were collected and placed in 2 mL cryotubes (Sarstedt, Nümbrecht, Germany). All samples were collected in RNALater preservative inactivation solution (Qiagen, Hilden, Germany), stored at 4 °C, then transported to and tested in South Africa at the National Institute for Communicable Diseases (NICD) and University of Pretoria. Permits were obtained from the Rwanda Development Board/Tourism & Conservation and animal ethics was obtained from the Animal Ethics Committee, University of Pretoria. 

### 2.2. RNA and DNA Extraction

For viral RNA detection, RNA was extracted from kidney, spleen, urine, fecal, rectal and intestinal samples (Table S1). RNA from kidney (*n* = 6), spleen (*n* = 5) and urine (*n* = 13), were extracted using the TRIzol reagent (Invitrogen, Carlsbad, CA, USA), and fecal material/swabs (*n* = 99), rectal and/or intestinal samples (*n* = 8) (Table S1) were extracted using the Duet RNA/DNA extraction kit (Zymoresearch, CA, USA) from samples homogenized in 300 µL of phosphate buffered saline (Lonza, Basel, Switzerland). Both extraction methods were performed according to the manufacturer’s instructions without deviations.

### 2.3. Morphological and Molecular Host Species Identification

DNA was extracted using the DNeasy Blood & Tissue Kit (Qiagen, Hilden, Germany) from heart tissues. Confirmation of species identification of bats, in which viral RNA was detected, was performed by amplifying the cytochrome b (cyt *b*) or cytochrome oxidase one (COI) gene region and determining the DNA sequence. Selection of cytochrome region for amplification was based on availability of credible comparative sequences in public databases (NCBI Genbank and BOLD). Previously reported PCR primers targeting these two regions were used or modified [39,40,41]. For the cyt *b* gene amplification, 1× DreamTaq buffer^TM^ (10×, ThermoFisher Scientific, Waltham, MA, USA), 2 µL Cyt *b*-forward modified primer (10 mM, Integrated DNA technologies, Coralville, IA, USA) (5’-CGA AGC TTG ATA TGA AAA ACC ATC GTT-3’), 2 µL Cyt *b*-reverse modified primer (5’-TGT AGT TRT CWG GGT CHT CTA-3’) (10 mM, Integrated DNA Technologies, USA), 1 µL dNTPs mix (10 mM, Invitrogen, Carlsbad, CA, USA), 0.25 µL of DreamTaq polymerase (5 U/µL, Thermo Scientific, USA), and nuclease-free water (Ambion, Foster City, CA, USA) to a final volume of 45 µL was prepared. A volume of 5 µL of extracted DNA was added to the reaction and incubated in a SimpliAmp automated thermal cycler (Thermofisher Scientific, Waltham, MA, USA) at 94 °C for 2 min; 45 cycles of 94 °C for 30 s, 50 °C for 30 s and 72 °C for 90 s; and 72 °C for 10 min. Amplification of the partial COI gene was done using the Folmer-LCO1490 forward (5’-GGT CAA CAA ATC ATA AAG ATA TTG G-3’) and Folmer-HCO2198 reverse (5’-TAA ACT TCA GGG TGA CCA AAA AAT CA-3’) primers as described for the cyt *b* gene with an annealing temperature of 48 °C.

Following the PCR analysis, reactions were subjected to agarose gel electrophoresis on a 1.5% agarose gel (Lonza, Basel, Switzerland) and PCR amplicons were gel-purified using the Wizard^®^ SV Gel DNA clean-up system (Promega, Madison, WI, USA) according to the manufacturer’s instructions and without deviation. All amplicons were subjected to Sanger sequencing for both the forward and reverse reactions on an ABI 3100 DNA sequencer (AE Applied Biosystems) at the sequencing facility of the University of Pretoria. Host gene sequences were subsequently compared to bat sequences available in the public domain (on the NCBI GenBank and BOLD databases), results were interpreted and compared with the respective morphological field identifications.

### 2.4. PCR Amplification of Viral Targets

#### 2.4.1. Coronaviruses

Fecal, rectal and/or intestinal samples from 101 bats (Table S1) were extracted and analysed for coronavirus RNA. Complementary DNA (cDNA) was prepared using 100 ng random hexamers (IE HPLC Purified, Integrated DNA Technologies, Coralville, IA, USA) with 200 U Superscript IV Reverse transcriptase (Thermo Scientific, Waltham, MA, USA). Additionally, cDNA was treated with 2 U RNase H (Thermo Fisher Scientific) incubation at 37 °C for 20 min and inactivated at 65 °C for 10 min. Presence of coronavirus RNA was detected with a coronavirus genus-specific hemi-nested RT-PCR assay which targets the RNA dependent RNA polymerase (RdRp) gene for amplification as described in Geldenhuys et al. [27]. As the hemi-nested RT-PCR assay produced only short amplicons (approximately 260 bp), the RdRp-grouping unit (RGU) assay was used to extend the sequenced region of the identified betacoronaviruses to 820 bp [16]. A hemi-nested RT-PCR assay was performed using the randomly primed cDNA prepared as well as forward primers from Drexler et al. [16] (SP3080 5’-CTT CTT CTT TGC TCA GGA TGG CAA TGC TGC-3’ and SP3195 5’-ATA CTT TGA TTG TTA CGA TGGT GGC TG-3’) in combination with a reverse primer (P1Beta_Rev2016 5’-CAT CRT CAS DIA RDA TCA TCAT-’3) from the Geldenhuys et al. [27] assay. Assay conditions from Geldenhuys et al. [27] were used with modifications to cycling conditions including longer annealing and extension cycles (45 cycles of 94 °C for 30 s, 42 °C for 60 s and 72 °C for 80 s). Agarose gel electrophoresis and purification of all PCR products were performed as previously described for molecular host species identification.

#### 2.4.2. Paramyxoviruses

Kidney from insectivorous bats, spleen from frugivorous bats and urine from both groups (*n* = 24; Table S1) were tested with the use of two broadly-reactive assays targeting the *Avula-Rubulavirinae* (AR) sub-families, and the *Respiro-Morbilli-Henipvirus* (RMH) genera. For both assays, published primers targeting the conserved polymerase (L) gene [42] were used in combination with adapted two-step hemi-nested RT-PCR protocols. Samples were tested with the AR assay as previously described [37]. For the RMH assay, the samples were tested as previously described [37], with minor variations in the protocol. For the first-round PCR, 25 mM MgCl_2_ (ThermoFisher Scientific, Waltham, MA, USA) was added and the nuclease-free water (Ambion, Foster City, CA, USA) was adapted for a final reaction volume of 50 µL. All cycling conditions and the protocol for the hemi-nested PCR remained the same as for AR. Agarose gel electrophoresis and purification of all PCR products were performed as previously described for molecular host species identification.

### 2.5. Sequencing and Phylogenetic Analysis

Sequencing was performed as previously described for molecular host species identification. Sequences were viewed, edited and a consensus generated using the BioEdit sequence alignment editor software version 7.2.5 [43]. CIPRES was used for ClustalX alignments, determining the best DNA substitution model for nucleotide sequence analysis using the jModelTest software and for constructing Bayesian phylogenies using the BEAST version 1.8 software [44,45,46]. Bayesian MCMC chains were set to 20 million iterations, sampling every 2000 steps for optimal ESS scores. Output files were visually inspected to check for convergence using the Tracer software version 1.7 [47]. The final phylogenies were constructed in TreeAnnotator with a burn-in value of 10%. For visualization and manipulation of the phylogenetic tree, the FigTree version 1.4.2 software was used. Pairwise similarities between sequences were analysed in MEGA X with complete deletion [48].

## 3. Results and Discussion

**Figure 2:**
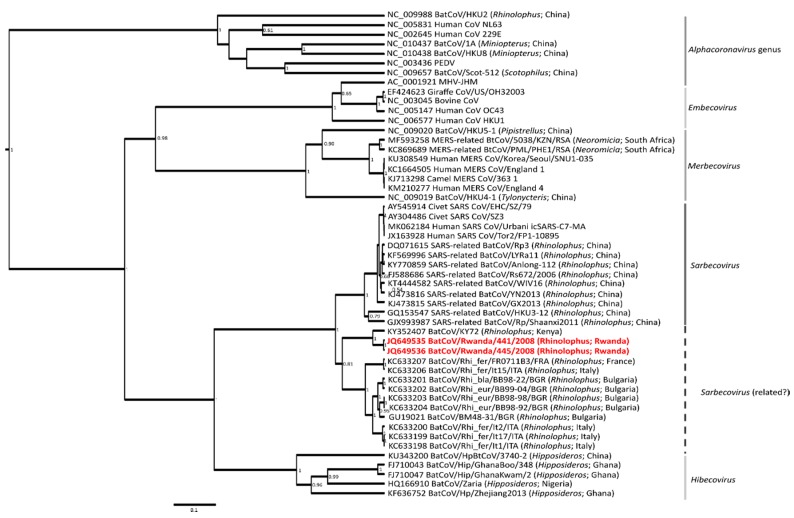
Bayesian phylogeny constructed based on a trimmed 780 bp sequence region of the RdRp gene of the coronavirus genome. The general time reversible model using gamma and invariant sites was determined using the jModelTest software version 2.1.6. The phylogeny was constructed using the BEAST version 1.8. Posterior probabilities of >0.5 are indicated at internal nodes. Subgenus and genus designations are indicated on the right, and sequences from this study are in red.

In total, samples from 101 bats constituting five genera were tested for coronaviruses (Table 1 and Table S1). Of these, two samples contained coronavirus RNA, originating from two individuals of the *Rhinolophus* genus. Barcoding and molecular identification confirmed the host species to be *Rhinolophus clivosus* (Table S1). Coronavirus sequences were extended to 820 bp with RGU assay primer sets [16]. The two sequences (*Rh*-BtCoV/441/Rwanda/08 and *Rh*- BtCoV/445/Rwanda/08) share 99.9% nucleotide identity; pairwise similarities and phylogenetic analysis group these sequences with other lineage B betacoronaviruses (Figure 2). The closest relative to the Rwandan betacoronavirus was reported from Kenya, BtCoVKY72 (Tao et al. unpublished; Genbank accession number KY352407.1), though the *Rhinolophus* species is not specified. The sequences share very close sequence similarities (95.5% nucleotide identity and 100% amino acid identity), suggesting that similar betacoronaviruses may be harbored by both Kenyan and Rwandan *Rhinolophus* bats. Full genome comparisons will be able to determine if Kenyan and Rwandan *Rhinolophus* bats are infected by the same betacoronavirus species. Within the analyzed conserved RdRp gene segment, this Rwandan *Rhinolophus* betacoronavirus also shares pairwise similarities of 88% nucleotide identity (98.9% amino acid identity) to the Bulgarian betacoronavirus *Rh*-BtCoV/BM48-31/BGR/08 [16], as well as close similarities (86.1–86.4% nucleic acid similarity and 98.9–99.2% amino acid identity) to Asian *Rhinolophus* SARS-related coronaviruses such as SARSr-*Rh*-BtCoV/Rp3 and SARSr-Rh-BtCoVWIV16 [18,49]. Other coronavirus surveillance in Rwanda, and surrounding countries such as Uganda and Tanzania also report SARS-related coronaviruses from the *Rhinolophus* genus [11]. Unfortunately, the sequences cannot be compared as an assay targeting a different conserved peptide of the RdRp gene was used [22]. 

**Figure 3:**
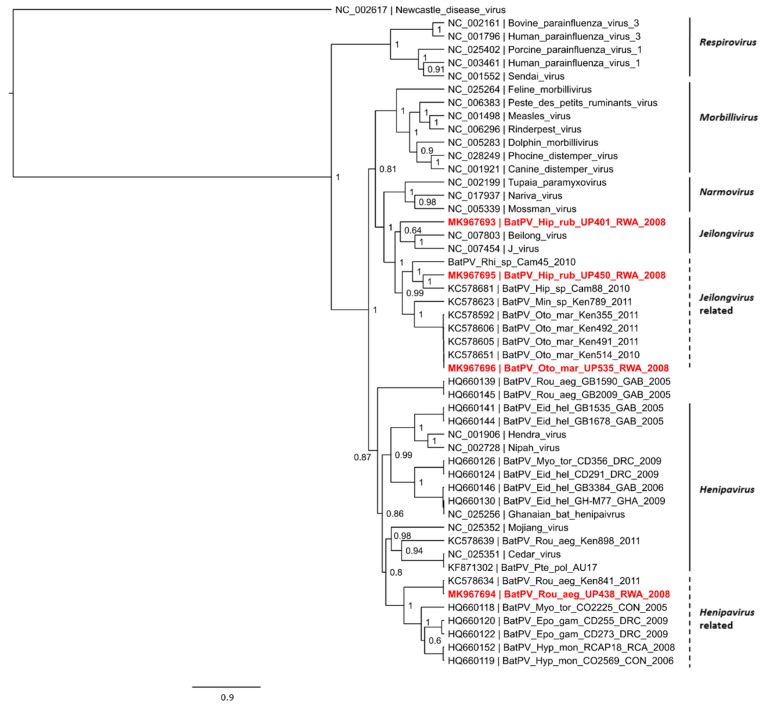
Phylogeny of the 439 nucleotide length sequences of the polymerase (L) gene of paramyxoviruses detected in bats from Rwanda using *Respiro-Morbilli-Henipavirus* genus-specific primers. Bayesian phylogenetic analysis was performed using the general time reversible model using the gamma and invariant site substitution model. A proportional tree representation is provided. Newly detected sequences from this study are indicated in bold red. Newcastle disease virus was selected as the outgroup.

Twenty-four samples from 23 bats were tested for paramyxovirus RNA (Table S1), none of which tested positive with the *Avula-Rubulavirinae* (AR) assay. An overall percentage positivity for paramyxovirus RNA, detected using the *Respiro-Morbilli-Henipavirus* (RMH) assay, was found to be 16.6% (*n* = 4). Three of the viral sequences were detected in the insectivorous bat species *Hipposideros ruber* and *Otomops martiensseni,* while one other sequence was detected in the frugivorous bat species *Rousettus aegyptiacus.* Host identification of positive samples was confirmed using molecular analysis (Table S1). Phylogenetic analysis of the sequences indicated that the insectivorous bat-borne viral sequences grouped with the *Jeilongvirus* genus as well as in a *Jeilongvirus*-related clade (Figure 3). One of the *H. ruber* sequences (BatPV/Hip_rub/UP401 /RWA/2008) described from this study potentially groups within the *Jeilongvirus* genus. The second *H. ruber*-derived viral sequence (BatPV/Hip_rub/UP450/RWA/2008) grouped with a paramyxoviral sequence detected in a bat from the same genus sampled in Cameroon in 2010, however, was not identical. The detection of two diverse viruses from bats of the same species and same population has previously also been reported in insectivorous bats sampled in other African countries [36]. These observations can in part be explained by the generation of viral quasi-species populations due to the high mutation rate of RNA viruses as a consequence of RNA proofreading deficiency of the RNA dependent RNA polymerase [50]. A larger pool of diverse viruses within a bat population and the co-roosting of several cave-dwelling bat species may facilitate viral sharing between different bat species [51]. However, ongoing biosurveillance in these cave-dwelling bat species will be required before active viral sharing can be shown. The paramyxoviral sequence detected in the *O. martiensseni* bat (BatPV/Oto_mar/UP535/RWA/2008) was near identical to the viral sequences previously described from several individuals of the same species sampled in Kenya in 2011 [36]. These sequences shared a 99.3% similarity on both nucleotide and amino acid level. The *R. aegyptiacus*-derived viral sequence (BatPV/Rou_aeg/UP438/RWA/2008) grouped within a *Henipavirus*-related clade and was near identical to a paramyxoviral sequence detected in the same host species previously reported from Kenya [36]. Sequence similarity shared between these two sequences was found to be 98.1% and 98.7% on nucleotide and amino acid level, respectively.

To our knowledge, this study reports on the first evidence of paramyxovirus RNA in bats from Rwanda. Two of the four viral sequences detected in *H. ruber*, were not closely related to the paramyxovirus sequences previously reported (sharing nucleotide and amino acid similarities of less than 80% and 83.5%, respectively) and might represent novel viral species. However, a more rigorous analysis with variable genes such as the fusion and hemagglutinin gene will be required before putative species can be inferred. As observed in previous studies, viral sequences from frugivorous bats were mostly found to belong to the *Henipavirus* genus or a related clade, while insectivorous bat-associated viral sequences have been linked to other genera including *Morbilli*- and *Jeilongvirus* [36]. This observation was again reflected in the current study. 

The detection of highly similar viral sequences from bats in Rwanda and Kenya, which are more than 1000 km apart, can be explained by either the phenomena of metapopulations or the hypothesis of co-evolution of paramyxoviruses with their bat hosts [3,52,53]. RNA viruses have an exceptionally high mutation rate commonly associated with quasi-species populations and with the potential to cross the species barrier, among others. This is evident in the high diversity of paramyxoviruses described to date and the wide host range associated with these viruses [3,28,36,54]. Additionally, due to the emergence of SARS, MERS and SADS, it is widely accepted that coronaviruses are capable of readily adapting to new hosts [5]. The Rwandan caves are considered an ecotourism site and guano is also mined on a small scale, providing an ideal bat-human interface. Several insectivorous bat species co-roost with the Egyptian fruit bats in these caves and future studies should investigate viral sharing. The Egyptian fruit bat also uses these caves as a maternity roost and studies have shown that increased viral shedding is linked to reproductive cycles [37]. Longitudinal biosurveillance studies can therefore identify high risk periods in the future. As such, the detection of a SARS-related bat coronavirus potentially circulating within the *Rhinolophus* population and a *Henipavirus*-related paramyxovirus in *R. aegyptiacus* in the Ruhengeri region may merit further investigation to determine exposure, and the potential for spill-over events to occur. To date, emphasis of paramyxovirus surveillance has mostly been placed on fruit bats, the *Henipavirus* genus and related viruses due to the association of other henipavirus species with zoonotic events [30,31]. However, research regarding the zoonotic potential of the insectivorous bat-associated viruses is still lacking. One major aim regarding surveillance of wildlife populations is to identify potential zoonotic agents and to evaluate any threat to the public as well as domestic animal health. Though these bat-borne viruses are unlikely to pose a significant threat, it still merits continued monitoring of the chiropteran species within these caves as well as mammalian species that inhabit the surrounding area.

For countries where the bat-human interface is more pronounced, as a result of ecotourism, guano mining or bat hunting and consumption, surveillance is key to identify the diversity of viruses present and their potential host species. Longitudinal and well-structured surveillance programmes would better characterize the circulation and shedding periods of the paramyxo- and betacoronaviruses within the Ruhengeri cave system. Such information would be most valuable towards well-considered zoonotic disease risk assessments and mitigation strategies. 

## Figures and Tables

**Table 1:** Bat-associated paramyxo- and coronavirus RNA detected from bats in the Ruhengeri caves in Rwanda.

Bat Species	Paramyxovirus Positive/Total	Coronavirus Positive/Total	Ruhengeri Site
*Hipposideros ruber* (Noack’s roundleaf bat)	**2/3**	0/2	Cave 1
*Otomops martiensseni* (Large eared free-tailed bat)	**1/4**	0/15	Cave 1
*Rousettus aegyptiacus* (Egyptian Rousette fruit bat)	**1/14**	0/72	Cave 1
*Rhinolophus* spp. (Horseshoe bat)	0/2	**2/7**	Cave 2
*Epomophorus* spp. (Epauletted fruit bat)	-	0/5	Cave 1
**Total**	**4/23**	**2/101**	

Boldface indicates positive samples.

## Supplementary Materials

The following are available online at https://www.mdpi.com/2414-6366/4/3/99/s1, Table S1: Bats collected and tested in this study.
