# Supplementary material for: Paramyxo- and Coronaviruses in Rwandan Bats
Source: Trop Med Infect Dis. 2019 Jul 2;4(3):99. doi: 10.3390/tropicalmed4030099 (PMC6789848; doi:10.3390/tropicalmed4030099)
Supplement: Supplementary file 1 [file tropicalmed-04-00099-s001.pdf]

Supplementary Table S1: Bat samples collected in Ruhengeri, Rwanda and tested for corona- and paramyxovirus RNA.

**Laboratory number**	**Date collected**	**Host**	**Voucher**	**Locality**	**SEX**	**Sample type tested**	**Paramyxovirus PCR***		**Coronavirus PCR**	**NCBI GenBank accession number (viral sequence)**	**Barcoding region**	
							**RMH**	**AR**			**Region**	**NCBI GenBank accession**
UP 401	10-Dec-08	*Hipposideros ruber*[#]	Yes	Cave 1	M	Kidney/ fecal swab	**Positive**	Negative	Negative	MK967693	CytB	MK984212
UP 402	10-Dec-08	*Rousettus aegyptiacus*	No	Cave 1	M	Fecal	-	-	Negative	-	-	-
UP 403	10-Dec-08	*Rousettus aegyptiacus*	No	Cave 1	M	Fecal swab	-	-	Negative	-	-	-
UP 404	10-Dec-08	*Rousettus aegyptiacus*	No	Cave 1	M	Fecal swab	-	-	Negative	-	-	-
UP 405	10-Dec-08	*Rousettus aegyptiacus*	No	Cave 1	F	Fecal swab	-	-	Negative	-	-	-
UP 406	10-Dec-08	*Rousettus aegyptiacus*	No	Cave 1	M	Fecal swab	-	-	Negative	-	-	-
UP 407	10-Dec-08	*Rousettus aegyptiacus*	No	Cave 1	M	Fecal swab	-	-	Negative	-	-	-
UP 408	10-Dec-08	*Rousettus aegyptiacus*	No	Cave 1	M	Fecal swab	-	-	Negative	-	-	-
UP 409	10-Dec-08	*Rousettus aegyptiacus*	No	Cave 1	F	Urine/ fecal swab	Negative	Negative	Negative	-	-	-
UP 410	10-Dec-08	*Rousettus aegyptiacus*	No	Cave 1	M	Fecal swab	-	-	Negative	-	-	-
UP 411	10-Dec-08	*Rousettus aegyptiacus*	No	Cave 1	F	Fecal swab	-	-	Negative	-	-	-
UP 417	10-Dec-08	*Rousettus aegyptiacus*	No	Cave 1	M	Fecal	-	-	Negative	-	-	-
UP 418	10-Dec-08	*Rousettus aegyptiacus*	No	Cave 1	F	Urine/ fecal swab	Negative	Negative	Negative	-	-	-
UP 419	10-Dec-08	*Rousettus aegyptiacus*	No	Cave 1	F	Fecal swab	-	-	Negative	-	-	-
UP 421	10-Dec-08	*Rousettus aegyptiacus*	Yes	Cave 1	M	Spleen/ fecal swab	Negative	Negative	Negative	-	-	-
UP 423	10-Dec-08	*Rousettus aegyptiacus*	No	Cave 1	M	Fecal swab	-	-	Negative	-	-	-
UP 424	10-Dec-08	*Rousettus aegyptiacus*	No	Cave 1	F	Urine/ fecal swab	Negative	Negative	-	-	-	-
UP 426	10-Dec-08	*Rousettus aegyptiacus*	No	Cave 1	M	Fecal	-	-	Negative	-	-	-
UP 433	11-Dec-08	*Rousettus aegyptiacus*	No	Cave 1	M	Fecal	-	-	Negative	-	-	-
UP 438	10-Dec-08	*Rousettus aegyptiacus*	Yes	Cave 1	F	Spleen/ Intestine	**Positive**	Negative	Negative	MK967694	COI	MK982175
UP 439	13-Dec-08	*Rhinolopus sp.*	No	Cave 2	F	Fecal	-	-	Negative	-	-	-

UP 441	13-Dec-08	*Rhinolopus clivosus*[#]	Yes	Cave 2	M	Kidney/ fecal	Negative	Negative	**Positive**	JQ649535	COI/ CytB	MK982176, MK984210
UP 442	13-Dec-08	*Rhinolopus sp.*	No	Cave 2	F	Fecal	-	-	Negative	-	-	-
UP 443	13-Dec-08	*Rhinolopus sp.*	No	Cave 2	F	Fecal	-	-	Negative	-	-	-
UP 444	13-Dec-08	*Rhinolopus sp.*	No	Cave 2	F	Urine/ fecal	Negative	Negative	Negative	-	-	-
UP 445	13-Dec-08	*Rhinolopus clivosus*[#]	No	Cave 2	M	Fecal	-	-	**Positive**	JQ649536	COI	MK982177
UP 446	13-Dec-08	*Rhinolopus sp.*	No	Cave 2	F	Fecal	**-**	-	Negative	-	-	-
UP 448	13-Dec-08	*Otomops martiensseni*	No	Cave 2	M	Urine	Negative	Negative	‘-	-	-	-
UP 450	13-Dec-08	*Hipposideros ruber*[#]	Yes	Cave 1	M	Kidney, Urine/ fecal, intestine	**Positive**	Negative	Negative	MK967695	CytB	MK984211
UP 452	13-Dec-08	*Otomops martiensseni*	No	Cave 1	M	Fecal	-	-	Negative	-	-	-
UP 453	13-Dec-08	*Otomops martiensseni*	Yes	Cave 1	F	Kidney/ intestine	Negative	Negative	Negative	-	-	-
UP 455	13-Dec-08	*Otomops martiensseni*	No	Cave 1	M	Fecal	-	-	Negative	-	-	-
UP 463	13-Dec-08	*Otomops martiensseni*	No	Cave 1	M	Fecal	-	-	Negative	-	-	-
UP 476	10-Dec-08	*Rousettus aegyptiacus*	No	Cave 1	M	Fecal	-	-	Negative	-	-	-
UP 477	10-Dec-08	*Epomophorous sp.*	No	Cave 1	F	Fecal swab	-	-	Negative	-	-	-
UP 478	10-Dec-08	*Epomophorous sp.*	No	Cave 1	M	Fecal swab	-	-	Negative	-	-	-
UP 479	10-Dec-08	*Epomophorous sp.*	No	Cave 1	M	Fecal swab	-	-	Negative	-	-	-
UP 480	10-Dec-08	*Rousettus aegyptiacus*	No	Cave 1	M	Fecal	-	-	Negative	-	-	-
UP 481	10-Dec-08	*Rousettus aegyptiacus*	No	Cave 1	M	Fecal swab	-	-	Negative	-	-	-
UP 482	10-Dec-08	*Rousettus aegyptiacus*	No	Cave 1	M	Fecal swab	-	-	Negative	-	-	-
UP 483	10-Dec-08	*Rousettus aegyptiacus*	No	Cave 1	F	Fecal swab	-	-	Negative	-	-	-
UP 486	10-Dec-08	*Rousettus aegyptiacus*	No	Cave 1	M	Urine/ fecal	Negative	Negative	Negative	-	-	-
UP 487	10-Dec-08	*Rousettus aegyptiacus*	No	Cave 1	M	Fecal swab	-	-	Negative	-	-	-
UP 488	10-Dec-08	*Rousettus aegyptiacus*	No	Cave 1	M	Fecal swab	-	-	Negative	-	-	-
UP 489	10-Dec-08	*Rousettus aegyptiacus*	No	Cave 1	F	Fecal	-	-	Negative	-	-	-
UP 490	10-Dec-08	*Rousettus aegyptiacus*	No	Cave 1	M	Fecal swab	-	-	Negative	-	-	-
UP 491	10-Dec-08	*Rousettus aegyptiacus*	No	Cave 1	M	Fecal	-	-	Negative	-	-	-
UP 494	10-Dec-08	*Rousettus aegyptiacus*	No	Cave 1	M	Fecal	-	-	Negative	-	-	-

UP 497	10-Dec-08	*Rousettus aegyptiacus*	No	Cave 1	F	Fecal	-	-	Negative	-	-	-
UP 498	10-Dec-08	*Rousettus aegyptiacus*	No	Cave 1	F	Urine/ fecal	Negative	Negative	Negative	-	-	-
UP 499	10-Dec-08	*Rousettus aegyptiacus*	No	Cave 1	F	Fecal swab	-	-	Negative	-	-	-
UP 500	11-Dec-08	*Rousettus aegyptiacus*	Yes	Cave 1	F	Spleen/ fecal, rectum/ intestine	Negative	Negative	Negative	-	-	-
UP 501	11-Dec-08	*Rousettus aegyptiacus*	No	Cave 1	F	Fecal	-	-	Negative	-	-	-
UP 502	11-Dec-08	*Rousettus aegyptiacus*	Yes	Cave 1	M	Spleen/ fecal, intestine	Negative	Negative	Negative	-	-	-
UP 505	11-Dec-08	*Rousettus aegyptiacus*	No	Cave 1	M	Fecal swab	-	-	Negative	-	-	-
UP 506	11-Dec-08	*Rousettus aegyptiacus*	No	Cave 1	F	Fecal swab	-	-	Negative	-	-	-
UP 507	11-Dec-08	*Rousettus aegyptiacus*	No	Cave 1	M	Fecal swab	-	-	Negative	-	-	-
UP 508	11-Dec-08	*Rousettus aegyptiacus*	Yes	Cave 1	F	Kidney / fecal swab	Negative	Negative	Negative	-	-	-
UP 509	11-Dec-08	*Rousettus aegyptiacus*	No	Cave 1	-	Fecal swab	-	-	Negative	-	-	-
UP 511	11-Dec-08	*Rousettus aegyptiacus*	No	Cave 1	M	Fecal	-	-	Negative	-	-	-
UP 512	11-Dec-08	*Rousettus aegyptiacus*	No	Cave 1	M	Fecal	-	-	Negative	-	-	-
UP 513	11-Dec-08	*Rousettus aegyptiacus*	No	Cave 1	F	Fecal swab	-	-	Negative	-	-	-
UP 514	11-Dec-08	*Rousettus aegyptiacus*	No	Cave 1	M	Fecal swab	-	-	Negative	-	-	-
UP 516	11-Dec-08	*Rousettus aegyptiacus*	No	Cave 1	M	Fecal	-	-	Negative	-	-	-
UP 517	11-Dec-08	*Rousettus aegyptiacus*	No	Cave 1	M	Urine	Negative	Negative	-	-	-	-
UP 519	11-Dec-08	*Rousettus aegyptiacus*	No	Cave 1	M	Fecal	-	-	Negative	-	-	-
UP 520	11-Dec-08	*Rousettus aegyptiacus*	No	Cave 1	F	Fecal	-	-	Negative	-	-	-
UP 525	11-Dec-08	*Rousettus aegyptiacus*	No	Cave 1	F	Fecal	-	-	Negative	-	-	-
UP 530	13-Dec-08	*Otomops martiensseni*	No	Cave 1	M	Fecal	-	-	Negative	-	-	-
UP 531	13-Dec-08	*Otomops martiensseni*	No	Cave 1	M	Urine/ fecal	Negative	Negative	Negative	-	-	-
UP 534	13-Dec-08	*Otomops martiensseni*	No	Cave 1	M	Fecal	-	-	Negative	-	-	-
UP 535	13-Dec-08	*Otomops martiensseni*	Yes	Cave 1	F	Kidney/ rectum, intestine	**Positive**	Negative	Negative	MK967696	COI	MK982178
UP 538	13-Dec-08	*Otomops martiensseni*	No	Cave 1	M	Fecal	-	-	Negative	-	-	-
UP 540	13-Dec-08	*Rousettus aegyptiacus*	No	Cave 1	F	Fecal	-	-	Negative	-	-	-
UP 543	13-Dec-08	*Rousettus aegyptiacus*	No	Cave 1	F	Urine	Negative	Negative	-	-	-	-

UP 582	10-Dec-08	*Epomophorous sp.*	No	Cave 1	M	Fecal	-	-	Negative	-	-	-
UP 583	10-Dec-08	*Epomophorous sp.*	No	Cave 1	F	Fecal	-	-	Negative	-	-	-
UP 584	10-Dec-08	*Rousettus aegyptiacus*	No	Cave 1	M	Fecal swab	-	-	Negative	-	-	-
UP 585	10-Dec-08	*Rousettus aegyptiacus*	No	Cave 1	M	Fecal	-	-	Negative	-	-	-
UP 586	10-Dec-08	*Rousettus aegyptiacus*	Yes	Cave 1	M	Spleen/ fecal swab	Negative	Negative	Negative	-	-	-
UP 587	10-Dec-08	*Rousettus aegyptiacus*	No	Cave 1	M	Fecal swab	-	-	Negative	-	-	-
UP 588	10-Dec-08	*Rousettus aegyptiacus*	No	Cave 1	F	Fecal	-	-	Negative	-	-	-
UP 589	10-Dec-08	*Rousettus aegyptiacus*	No	Cave 1	M	Fecal swab	-	-	Negative	-	-	-
UP 590	10-Dec-08	*Rousettus aegyptiacus*	No	Cave 1		Urine	Negative	Negative	-	-	-	-
UP 591	10-Dec-08	*Rousettus aegyptiacus*	No	Cave 1	M	Fecal	-	-	Negative	-	-	-
UP 592	10-Dec-08	*Rousettus aegyptiacus*	No	Cave 1	F	Fecal	-	-	Negative	-	-	-
UP 593	10-Dec-08	*Rousettus aegyptiacus*	No	Cave 1	M	Fecal swab	-	-	Negative	-	-	-
UP 594	10-Dec-08	*Rousettus aegyptiacus*	No	Cave 1	F	Fecal swab	-	-	Negative	-	-	-
UP 595	10-Dec-08	*Rousettus aegyptiacus*	No	Cave 1	F	Fecal	-	-	Negative	-	-	-
UP 596	10-Dec-08	*Rousettus aegyptiacus*	No	Cave 1	M	Fecal	-	-	Negative	-	-	-
UP 597	10-Dec-08	*Rousettus aegyptiacus*	No	Cave 1	M	Fecal swab	-	-	Negative	-	-	-
UP 598	10-Dec-08	*Rousettus aegyptiacus*	No	Cave 1	M	Fecal	-	-	Negative	-	-	-
UP 599	10-Dec-08	*Rousettus aegyptiacus*	No	Cave 1	M	Fecal swab	-	-	Negative	-	-	-
UP 600	11-Dec-08	*Rousettus aegyptiacus*	No	Cave 1	F	Fecal	-	-	Negative	-	-	-
UP 601	11-Dec-08	*Rousettus aegyptiacus*	No	Cave 1	M	Fecal swab	-	-	Negative	-	-	-
UP 602	11-Dec-08	*Rousettus aegyptiacus*	No	Cave 1	M	Fecal swab	-	-	Negative	-	-	-
UP 605	11-Dec-08	*Rousettus aegyptiacus*	No	Cave 1	F	Fecal	-	-	Negative	-	-	-
UP 606	11-Dec-08	*Rousettus aegyptiacus*	No	Cave 1	M	Fecal	-	-	Negative	-	-	-
UP 610	11-Dec-08	*Rousettus aegyptiacus*	No	Cave 1	F	Fecal	-	-	Negative	-	-	-
UP 611	11-Dec-08	*Rousettus aegyptiacus*	No	Cave 1	F	Fecal	-	-	Negative	-	-	-
UP 619	13-Dec-08	*Otomops martiensseni*	No	Cave 1	M	Fecal swab	-	-	Negative	-	-	-
UP 622	13-Dec-08	*Hipposideros sp.*	No	Cave 1	F	Urine	Negative	Negative	-	-	-	-

UP 624	13-Dec-08	*Otomops martiensseni*	No	Cave 1	M	Fecal	-	-	Negative	-	-	-
UP 625	13-Dec-08	*Otomops martiensseni*	No	Cave 1	M	Fecal	-	-	Negative	-	-	-
UP 626	13-Dec-08	*Otomops martiensseni*	No	Cave 1	M	Fecal	-	-	Negative	-	-	-
UP 627	13-Dec-08	*Otomops martiensseni*	No	Cave 1	M	Fecal	-	-	Negative	-	-	-
UP634	13-Dec-08	*Otomops martiensseni*	No	Cave 1	M	Fecal	-	-	Negative	-	-	-

* RMH: *Respiro-Morbilli-Henipavirus* assay; AR: *Avula-Rubulavirinae* assay. Samples positive are indicated in boldface. # indicated hosts confirmed with barcoding.
